# Therapeutic efficacy of artemether-lumefantrine for *Plasmodium vivax* infections in a prospective study in Guyana

**DOI:** 10.1186/1475-2875-11-347

**Published:** 2012-10-19

**Authors:** Daniel Eibach, Nicolas Ceron, Karanchand Krishnalall, Keith Carter, Guillaume Bonnot, Anne-Lise Bienvenu, Stéphane Picot

**Affiliations:** 1 Malaria Research Unit, CNRS UMR 5246, University Lyon 1, Faculty of Medicine, Lyon, France; 2 European Public Health Microbiology Training Programme (EUPHEM), European Centre for Disease Prevention and Control (ECDC), Stockholm, Sweden; 3 Pan-American Health Organization, Georgetown, Guyana; 4 Vector Control Services, Malaria Control Programme, Ministry of Health Guyana, Georgetown, Guyana; 5 Division of Disease Prevention and Control, Pan-American Health Organization, Washington, USA

**Keywords:** Malaria, Plasmodium vivax, Artemether-lumefantrine, Chloroquine, Guyana

## Abstract

**Background:**

In Guyana, chloroquine + primaquine is used for the treatment of vivax malaria. A worldwide increase of chloroquine resistance in *Plasmodium vivax* led to questioning of the current malaria treatment guidelines. A therapeutic efficacy study was conducted using artemether-lumefantrine + primaquine against *P. vivax* to evaluate a treatment alternative for chloroquine.

**Methods:**

From 2009 to 2010, a non-controlled study in two hospitals in Guyana was conducted. A total 61 patients with *P. vivax* infection were treated with artemether-lumefantrine as a six-dose regimen twice a day for three days with additional 0.25 mg/kg/d primaquine at day 0 for 14 days. Clinical and parasitological parameters were followed on days 0,1,2,3,7,14 and 28 in agreement with WHO guidelines. *Plasmodium vivax* DNA from eight patients was analysed for *pvmdr1*, molecular marker of resistance.

**Results:**

Artemether-lumefantrine cleared 100% of parasites on day 1, but two patients (3%) had recurrence of parasites on day 28, suggesting relapse. No *pvmdr1* Y976F polymorphism was detected. The treatment regimen was well tolerated.

**Conclusions:**

In Guyana, artemether-lumefantrine represents an adequate treatment option against *P. vivax* when combined with primaquine. Availability of this alternative will be of great importance in case of emerging chloroquine resistance against *P. vivax.*

## Background

Approximately 216 million malaria cases were reported world-wide in 2010 [1]. Outside of Africa, the most widely distributed species is *Plasmodium vivax*, followed by *Plasmodium falciparum*[1]. In South America, 1–1.2 million cases of malaria are reported annually with *P. vivax* responsible for three quarters and *P. falciparum* for one quarter of cases [2].

Artemisinin-based combination therapy (ACT) has been and still is one of the main pillars of the treatment of malaria and is responsible for the reduction of malaria infections worldwide [3-7]. In recent years, reports from the border between Thailand and Cambodia [8,9] show resistance from *P. falciparum* to artemisinin-derivatives. Although in vitro resistance to artemether is reported for *P. falciparum* from South America [10], so far there is no evidence for treatment failures. Until now, only insufficient information on treatment outcomes with ACT for the treatment against *P. vivax* is available.

In Guyana, 42.3% of all malaria cases between 2005 and 2009 were caused by *P. falciparum* and 51.3% by *P. vivax*. The number of malaria cases declined rapidly until 2007, but increased slightly in 2009, with 93% of the population still being at risk of contracting malaria [1].

Anti-malarial drug policies in South American countries, including Guyana, use ACT for uncomplicated *P. falciparum* and chloroquine + primaquine for *P. vivax* infections.

Chloroquine resistance against *P. vivax* was first reported in 1989 from Papua New Guinea [11]. Since then various countries, including Myanmar, Turkey, Ethiopia, Vietnam, Indonesia, Korea and Madagascar reported treatment failures with chloroquine [1,12-16]. Thailand reported its first chloroquine resistant *P. vivax* cases in 2011 [17], while in India chloroquine treatment seems still to be effective [18]. Reports from Colombia, Brazil, and Guyana [19] indicate chloroquine resistance appearing in numerous regions of South America [20,21].

This study aims to assess artemether-lumefantrine + primaquine as an alternative treatment option for *P. vivax* malaria, as a decline in susceptibility towards chloroquine can be predicted for the coming years. In the light of poor access to diagnostic facilities and emerging chloroquine resistance it is important to test the efficacy of artemether-lumefantrine as a common malaria treatment in Guyana. In remote areas, WHO recommends the use of rapid tests for malaria diagnosis. While several of these commercially available tests showed very good performances, their sensitivity and specificity for vivax Malaria is generally lower than for falciparum malaria. Since malaria species diagnosis could be uncertain, the issue of treatment efficacy of a single common treatment for all circulating plasmodium species had to be addressed.

Further the study aims to screen for *pvmdr1* single-nucleotide polymorphism at codon Y976F, suspected to be involved in *P. vivax* resistance to chloroquine [22].

## Methods

### Patient enrolment, treatment and follow-up procedures

A prospective multicentre study was conducted in 2009/2010 for artemether-lumefantrine + primaquine against *P. vivax*. *S*amples have been collected at two sites, the Malaria clinic Georgetown, situated in the administrative district 4 (Demerara-Mahaica; pop. 310.320), and at Port Kaituma Hospital in region 1 (Barima-Waini; pop. 24.275) of Guyana.

Included were all patients older than six month, weighing more than 10 kg and with a parasitological confirmed *P. vivax* mono*-*infection.

Patients were excluded from the study, if any of the following occurred: (i) presence of general danger signs in children aged under five years or signs of severe falciparum malaria according to the definitions of WHO [23], (ii) presence of severe malnutrition (defined as a child <5 years of age with a weight-for-height z-score < −3, with symmetrical oedema involving at least the feet or with a mid-upper arm circumference <110mm), (iii) history of hypersensitivity reactions or contraindications to any of the medications being tested, (iv) a positive pregnancy test or breastfeeding, (v) unable to or unwilling to take contraceptives, (vi) women between 12–26 years old in order to avoid potentially pregnant participants, (vii) use of anti-malarial drugs outside of the study protocol or regular medication, which may interfere with anti-malarial pharmacokinetics, (viii) detection of mixed malarial infections during follow-up, (ix) presence of febrile conditions due to diseases other than malaria, (x) withdrawal of consent, (xi) loss to follow-up, (xii) protocol violation, or (xiii) death due to a non-malaria illness.

All study participants were treated with the treatment regimen used for *P. falciparum* infections, according to the national drug policy. They received artemether-lumefantrine (Novartis, batch number F1375, expiry date: 01.01.2011) as a six-dose regimen twice a day for three days and additional 0.25 mg/kg/d primaquine (NEW GPC INC Guyana, batch number B10392, expiry date 01.09.2011) for 14 days. The treatment dose was re-administered, if patients vomited during 30 minutes after administration. Patients repeatedly vomiting after their first dose of study medication were excluded from the study. The three-day artemether-lumefantrine treatment was directly supervised on site, while the intake of primaquine was not directly observed. The study participants were not checked for glucose-6-phosphate dehydrogenase (G6PD) deficiency prior to primaquine administration, as its prevalence in Guyana is assumed to be low; however accurate information on this deficiency in Guyana is scarce. When participants presented symptoms of haemolytic anaemia (jaundice, dark urine, abdominal pain, back pain, lowered haemoglobin level) during the follow-up visits, the primaquine treatment had to be stopped and they were excluded from the study.

### Outcome measures

Treatment outcomes were classified on the basis of the parasitological and clinical outcome according to the latest WHO guidelines [24], which classifies all patients as having early treatment failure, late clinical failure, late parasitological failure or an adequate clinical and parasitological response (ACPR). The ACPR was calculated for day 14 and day 28.

### Laboratory procedures

Clinical examination, including measurement of the axillary temperature and Giemsa staining of thick and thin blood films, was carried out on days 0, 1, 2, 3, 7, 14, 21, and 28. Thick and thin blood smears were prepared from finger sticks. After Giemsa staining, each slide was examined microscopically independently by two experienced technicians at the collection site and again at the Malaria Research Unit, University Lyon 1. The parasitaemia was recorded as number of asexual parasites counted per 200 white blood cells present in the thick smear. The thick-film was considered to be negative, if no parasite had been found in 100 high-power fields. On days 0, 7, 14, 21 and 28 blood was blotted onto filter paper during follow-up and stored for DNA analysis.

### DNA extraction and amplification

DNA was extracted from blood spots on filter paper with resin-based Instagene Matrix (Bio-Rad, Marnes la Coquette, France), as described before [25]. Genus-specific primers targeting the 18s rRNA were used to screen samples for the presence of Plasmodium. The identification of the parasite species was confirmed with species-specific primers (25), using LightCycler (Roche) real-time PCR. DNA was only available for a subset of eight *P. vivax* cases.

### *Plasmodium vivax mdr1* gene

Detection of SNPs in *pvmdr1* (Y976F) was performed on the eight *P. vivax* cases using a LightCycler system (Roche) and fluorescence resonance energy transfer technology. Primers and probes were designed and synthesized by TIB Molbiol (DNA Synthesis Service, Berlin, Germany). The polymerase chain reaction mixture and assay conditions were used as described in detail elsewhere [22,26,27].

### Ethical approval

The study protocol was reviewed and approved by the Ethics Committee of the Ministry of Health of Guyana. Informed written consent was provided by all patients or their parents/guardians before inclusion in the study.

## Results

### Study population

A total of 561 patients were screened for possible enrolment into the study and 74 (13.2%) patients met the inclusion criteria. The Georgetown hospital contributed 87.8% (n=65) of patients, 12.2% (n=9) were recruited at the Port Kaituma Hospital. Among the patients included, 90.5% were male, 9.5% female, with the majority of all participants (86.5%) being between 15–49 years old.

The different ethnicities of Guyana were representatively distributed with 47.3% from mixed ethnicities, 24.3% East Indians, 16.2% Amerindians, 10.8% Afroguyanes and 1.4% of other groups. In total, 98% of the study population originated from the three highest malaria endemic regions 1, 7 and 8. The patients presented with the following symptoms in the preceding 24h: (i) Fever 20.3%, (ii) vomiting 1.4%, (iii) diarrhoea 2.8%, (iv) problems with coordination 1.4%, (v) dizziness or fainting 4.1%. However the fever incidence will be underestimated due to frequent self-medication with antipyretic drugs. The mean parasitaemia counts were 3,920 parasites/μl ranging from a maximum of 29,100 parasites/μl to a minimum of 30 parasites/μl (Table 1).

**Table 1:** Demographic, parasitological and clinical features of patients enrolled in the study

	**n=74**
Age (median, range)	24 (5–57)
Male/female	67/7
baseline parasitaemia (mean, range, /μl)	3920 (30–29100)
baseline parasitaemia Group (/μl)	
0-1000	24 (32.4%)
1000-10000	44 (59.5%)
10000-100000	6 (8.1%)
>100000	0 (0%)
Baseline Temperature at admission (mean, range, °C)	36.6 (36.0-39.2)
Developed PV parasitaemia during follow-up	2 (2.7%)

**Figure 1:**
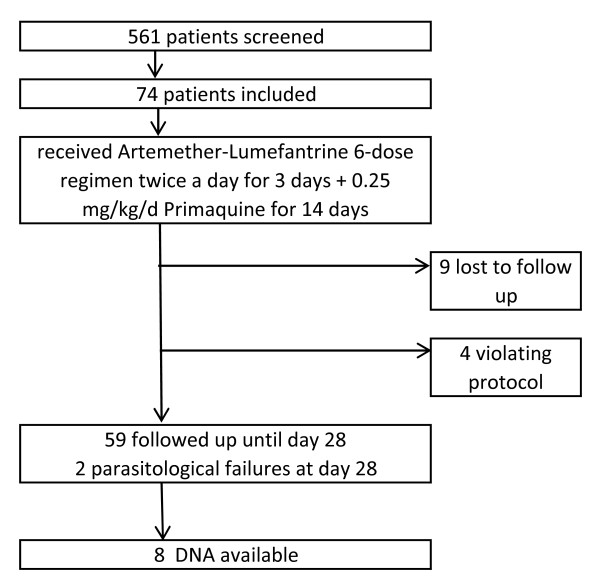
Flow chart of the study procedure.

Out of the 74 included patients, nine patients were lost for follow-up and four violated the protocol, resulting in 61 patients, which completed the study (Figure 1).

### Treatment study

For all patients receiving treatment fever abated and parasites were cleared at day 1. Two patients were found with parasitaemia at day 28 of the follow-up period, showing a parasite density of 487/μl and 780/μl respectively. The crude ACPR was 61/61 (100%) by day 14, decreasing to 59/61 (97%) by day 28. All patients tolerated the treatment well with very few side effects and no serious adverse events observed. No participants had to be excluded for haemolytic anaemia after primaquine administration.

In a subset of eight patients, the *P. vivax* mono-infection was PCR-confirmed. The others were microscopically confirmed by three independent readers. DNA from the parasites from the eight confirmed cases underwent screening for *pvmdr1* single-nucleotide polymorphism at codon Y976F. No mutations were found.

## Discussion

The study assessed artemether-lumefantrine + primaquine as a treatment option against *P. vivax*. Chloroquine and primaquine have been used worldwide against *P. vivax* as first-line drugs since 1946 and 1950 respectively. Reports from South-American countries such as Colombia and Brazil [20,21] on decreasing chloroquine efficacy against *P. vivax* and a change of drug policies from chloroquine to ACT for vivax infections in four countries (Solomon Islands, Vanuatu, PNG and Papua (Indonesia)) [1], led to the question for an alternative treatment option in Guyana. So far only a few studies have examined artemether-lumefantrine as a treatment option for *P. vivax* and even less studies looked at artemether-lumefantrine and primaquine.

All patients in the study cleared parasites on day 1, which makes artemether-lumefantrine an efficacious alternative for chloroquine in the treatment of vivax malaria. No severe side effects or adverse events were observed, similar to other studies, which show a good safety profile for artemether-lumefantrine alone [28-31] and for artemether-lumefantrine in combination with primaquine [30], when used for *P. vivax* infections. Drug interactions between the two drugs seem to be unlikely [32].

Two patients (2.7%) presented with parasitological failure at day 28. In one of those patients *P. vivax* was PCR-confirmed at day 28, with negative PCRs on day 7, 14 and 21. It can be assumed that the new parasitaemia derived from relapse, rather than recrudescence, although a new infection or a recrudescence cannot be ruled out completely since there is no evidence that genotyping of *P. vivax* parasites may help to distinguish these events [33]. Frequent relapses with artemether-lumefantrine have been described, due to the short half-life of lumefantrine [28]. However, a combination with a longer-acting partner drug, such as piperaquine would only delay the *P. vivax* relapse, but not prevent it. Lumefantrine on the other hand is less likely to induce resistance compared to long half-life drugs as chloroquine or piperaquine [34]. In a situation, where primaquine can be administered in a safe and compliant manner, primaquine is the ideal drug for preventing relapse. However, a concerning rate of 24.5% resistance to primaquine has been described in Brazil [35], which could be one reason for the two treatment failures, observed in the study. Another reason might be a poor compliance to the unsupervised primaquine treatment.

With recent reports clearly demonstrating severe *P. vivax* infections resembling the course of malign *P. falciparum* infections [36,37] the application of a sufficient therapy has to be ensured. Therefore the continuing surveillance of chloroquine efficacy is of great importance. Studying the history of anti-malarial drug resistance development worldwide, it is not hard to predict that we also will encounter frequent treatment failures due to chloroquine in the upcoming years in South America.

A drawback to this study is that only eight patients could be screened for *pvmdr1* single-nucleotide polymorphism and no mutations in codon Y976F were found. Low parasitaemia and sub-optimal sample storage conditions might partly explain the poor usefulness of the PCR method with these samples, while it usually works well with many other blood spot samples. No conclusions can be drawn on any resistance associated with *pvmdr1* polymorphism. One out of the two patients with treatment failure was tested for Y976F mutation, but was wild type. In total, 90% of the study participants were male. In Guyana, malaria affects many more males than females at a ratio of almost 4:1 (males 78.2% and females 21.8%). This reflects the fact, that the migrant populations, primarily miners, are by far the most affected group. In addition to that all women aged 12 to 26 were excluded, in order to avoid potential pregnant study participants. 60% of all reported malaria cases occur in the Amerindian population, to which 16.2% of the study participants belong [38].

A switch of drug policies in Guyana to artemether-lumefantrine + primaquine for *P. vivax* infections would establish a common treatment for both circulating *Plasmodium* species, which are *P. falciparum* and *P. vivax.* Artemether-lumefantrine is highly effective against *P. falciparum* in Guyana, based on a study conducted in 2007/2008 (National Malaria Control Program, unpublished data). A common treatment could result in a number of advantages. Reports from Thailand and Indonesia detected up to 23% of *P. falciparum* infections being wrongly diagnosed as *P. vivax*, leading to inappropriate treatment with chloroquine [39,40]. ACT has also shown to clear *P. vivax* more quickly than chloroquine [41]. Treating *P. falciparum* with artemether-lumefantrine + primaquine would have the advantage of also treating potential dormant *P. vivax* hypnozoites, which are thought to become activated due to *P. falciparum* infections, thereby explaining the high number of vivax infections after *P. falciparum* treatment [33].

Keeping chloroquine in the market, could lead to its administration against *P. falciparum* in the private sector. Disadvantages of a common treatment will be the higher price for ACT as well as a decreased impetus for laboratories and hospitals in performing a correct plasmodium species diagnostic. The prescription of primaquine to all malaria cases without prior testing for G6PD deficiency presents a potential risk for severe haemolytic anaemia in G6PD-deficient patients, although G6PD deficiency is an infrequent trait in Guyana.

## Conclusions

This study shows excellent efficacy for artemether-lumefantrine in combination with primaquine against *P. vivax* infections. The treatment efficacy for chloroquine for *P. vivax* must be under close surveillance in Guyana. Should resistance against *P. vivax* increase, the tested regimen with artemether-lumefantrine + primaquine will be a more than equal alternative and drug policies should be switched.

## Competing interests

The authors declare that they have no competing interests.

## Authors' contributions

DE contributed to molecular genetic studies, interpretation of results and the drafting of the manuscript. NC organized the collection of samples in Guyana and helped with the interpretation of results. KK and KC contributed to the study design and the interpretation of results. GB generated a portion of the molecular data. ALB contributed to the interpretation of the data and writing of the manuscript. SP contributed to the study design, interpretation of results and the writing of the manuscript. All authors read and approved the final manuscript.
